# Clinical Assessment of Intermittent Fasting With Ketogenic Diet in Glycemic Control and Weight Reduction in Patients With Type II Diabetes Mellitus: A Systematic Review and Meta-Analysis

**DOI:** 10.7759/cureus.30879

**Published:** 2022-10-30

**Authors:** Hany A Zaki, Haris Iftikhar, Abeer Abdalrubb, Nood Dhafi R Al-Marri, Mohammed Gafar Abdelrahim, Mohamed Fayed, Mohamed Abdelgadir M Elgassim, Mohamed A Elarref

**Affiliations:** 1 Emergency Medicine, Hamad Medical Corporation, Doha, QAT; 2 Endocrinology and Diabetes, Hamad Medical Corporation, Doha, QAT; 3 Anaesthesiology, Weill Cornell Medicine - Qatar, Doha, QAT; 4 Anesthesiology, Intensive Care and Perioperative Medicine, Hamad Medical Corporation, Doha, QAT

**Keywords:** very low-carbohydrate diet, ketogenic, diabetes mellitus type 2, glycated hemoglobin (hba1c), glycemic control, intermittent energy restriction, time-restricted fasting, intermittent fasting

## Abstract

Diabetes mellitus (DM) is a global epidemic causing significant morbidity and mortality. The most occurring DM is type 2 diabetes mellitus (T2DM) which has similar symptoms as type 1 diabetes mellitus (T1DM). However, it is less marked, making it difficult to diagnose during the early stages. The management of T2DM is usually based on weight and glycemic control, which can be achieved through dietary interventions such as intermittent fasting (IF) and the ketogenic diet (KD). Therefore, this systematic review and meta-analysis aim to demonstrate the role of IF and KD in glycemic and weight control among patients with T2DM.

Two methods, including an electronic database search through ScienceDirect, Google Scholar, PubMed, Scopus, Embase, and Web of Science, and a manual search were used to identify relevant studies published between 2000 and 2022. The search yielded 1299 articles, of which only 12 met the inclusion criteria. In addition, study quality appraisal was performed using Review Manager software (RevMan 5.4.1). The pooled results have shown that IF had a similar effect on HBA1c reduction as control interventions (standardized mean differences [SMD]: 0.36%; 95% CI; -0.37, 1.10; P = 0.33, I2 = 87%). Similarly, an insignificant difference in weight reduction between IF and control interventions was recorded (SMD: -1.05%; 95% CI; -2.29, 0.19; P = 0.10, I2 = 96%). On the other hand, KD significantly reduced body weight compared with control diets (SMD: -1.91 kg; 95% CI; -2.96 kg, -0.85 kg; P = 0.0004, I2 = 96%). Similarly, KD had a better effect on the HBA1c percentage reduction than control diets (SMD: -2.00%; 95% CI; -3.76, -0.25; P = 0.03, I2 = 97%). IF and KD have shown reductions in HBA1c and body weight among patients with T2DM. However, the interventions are subject to side effects and should be used with caution and under the supervision of a health professional.

## Introduction and background

Diabetes mellitus (DM) is a global epidemic causing significant morbidity and mortality. More recent statistics by the International Diabetes Federation (IDF) have shown that as of 2021, 537 million adults were living with diabetes, estimated to rise to 783 million by 2045 [1]. It is also reported that at least 6.7 million DM deaths were observed worldwide in 2021. DM has also contributed to an increased economic burden in most countries, with at least $966 billion being used in health expenditures by 2021. This expenditure was a 316% increase from the last 15 years [1]. This disease usually presents itself as either type 1 diabetes mellitus (T1DM), type 2 diabetes mellitus (T2DM), or gestational diabetes. T2DM, formerly known as non-insulin-dependent or adult-onset diabetes, usually results from the ineffective use of insulin by the body and is estimated to be prevalent in about 95% of people with diabetes [2]. Symptoms of T2DM are usually similar to those of T1DM; however, they are often less marked. Therefore, the disease is generally diagnosed late after onset when the complications have already presented themselves. Factors largely contributing to T2DM are usually overweight, obesity, and lack of physical activity. Therefore, the disease can primarily be managed by weight loss through a diet, preferably combined with physical activity. Various dietary plans, including low carbohydrate diets, ketogenic diets (KD), intermittent fasting (IF), Mediterranean diets, and low glycemic index diets, have been implemented in managing T2DM. IF involves repeatedly and intentionally interrupting energy consumption for a specific period. The main reason for the fasting regimens is usually to maximize the oxidation of fatty acids and ketone bodies rather than relying on glucose as a source of energy. Additionally, prolonged fasting contributes to more extended periods where insulin levels are low, assisting in weight loss and improved insulin sensitivity through hyperinsulinemia reduction [3,4]. Even though IF interventions have not been standardized, the commonly used regimens include time-restricted feeding, which involves feeding for a period between 4 and 8 hours/day and fasting for 16-20 hours, and the intermittent or short-term energy restriction achieved by very low-calorie diets (VLCD) whereby the calorie consumption is about 300-600 kcal/day. Evidence in previous scientific research also shows that IF can be harmful. For example, Cahill LE et al. [5] reported that time-restricted feeding, such as breakfast skipping, is associated with an increased risk of coronary heart disease, T2DM, and other adverse events. Similarly, it has been reported that for people with T2DM, skipping breakfast increases the risk of glycemic spikes than standardized lunch and dinner [6]. Based on this evidence, the benefit of IF in T2DM patients remains somewhat uncertain.

On the other hand, KD has gained interest as a quick and effective weight loss strategy. KD is usually described as a diet that consists of very-low carbohydrates and high fat to induce nutritional ketosis in patients. The induced ketosis, in turn, suppresses appetite in the patients; thus, it has been proposed as an effective measure for weight loss [7]. Previous studies have also reported that KD positively affects other health outcomes, such as improving glycemic control and reducing medication dosages [8,9]. Additionally, a prior review article comparing VLCD to normal carbohydrate diets reported improvements in short-term weight, reduced glycated hemoglobin, and a significant reduction of medications among overweight T2DM patients that adhered to VLCD protocol [10]. Even though the value of KD and IF on weight loss and glycemic control have been recorded in previous studies, their effects on patients with T2DM are still uncertain. Therefore, this study evaluated the role of KD and IF among T2DM patients. To understand the impact of the two diets, this review will identify their influence on body weight, BMI, and glycated hemoglobin (HBA1c). We hypothesize that both diets will significantly reduce BMI, body weight, and HBA1c.

## Review

Methodology

Protocol and Registration

This article followed the Prospective Register of Systematic Reviews (PROSPERO) database protocol and registration [11]. In addition, the manuscript preparation observed the Cochrane Collaboration procedures and reported as per the Preferred Reporting Items for Systematic Reviews and Meta-analyses (PRISMA) guidelines [12].

Eligibility Criteria

Analyzing every study based on the inclusion and exclusion criteria was assigned to two reviewers. Studies were included for review in the present study if they met the following criteria: articles written and published in English. This criterion was vital as it helped to avoid direct translation of scientific terms that could lose meaning and context, pieces that either made a comparison of KD or IF to other diets in T2DM patients, and studies with sufficient sample size, i.e., more than 10 patients. This specification was made to improve the statistical power of the present meta-analysis, articles whose outcomes included BMI, body weight, or HBA1c, and studies that included human subjects. On the other hand, studies were excluded based on the criteria outlined as follows; non-English articles, studies that included animal subjects, studies that did not evaluate either KD or IF, studies that compared either KD or IF in patients with other conditions or diseases, such as obesity, prediabetes and T1DM, systematic reviews and meta-analyses, letters to the editor, case reports, and abstracts without evidence of full articles.

Literature Search

A detailed search for randomized controlled trials and other primary studies was carried out in accordance with the PRISMA guidelines. The search was performed on five electronic databases: ScienceDirect, Google Scholar, PubMed, Scopus, Embase, and Web of Science. The Boolean operators “AND” and “OR” were used in conjunction with specific keywords for a detailed search strategy which was as follows: (ketogenic OR keto diets OR very low-carbohydrate diet OR very low-calorie diet) AND (intermittent fasting OR time-restricted fasting OR intermittent energy restriction) AND (glycemic control OR glycated hemoglobin OR HBA1c) AND (body weight OR body mass index OR BMI) AND (type 2 diabetes mellitus OR T2DM). A manual search was also conducted on the references of identified studies to supplement the search strategy. All query results were limited to a period between 2000 and 2022.

Data Extraction

The data extraction process was carried out by two reviewers who then compiled all the relevant data retrieved from the included studies. The main data extracted included Author ID, study design, country of origin, participants, intervention diet, control diet, and the main outcomes. The author ID was subdivided into the first author's surname and year of publication, while the participant characteristics were age, sample size, and sex. The main outcomes of the present study were glycemic control and weight loss. Glycemic control was obtained by analyzing HBA1c outcomes, while weight loss was analyzed by recording the BMI and body weight outcomes. Any discrepancies in recorded data were resolved by debating between the reviewers or consulting a third reviewer.

Quality Assessment

**Figure 1:**
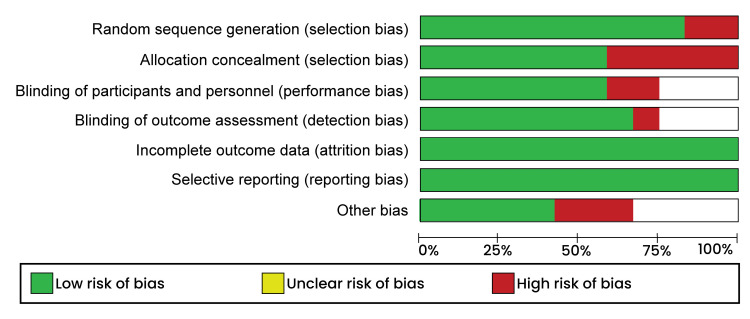
Risk of bias graph.

Study quality assessment was carried out using the criteria outlined in the Cochrane handbook for systematic review and was performed using Review Manager software (RevMan 5.4.1). During the assessment, the reviewers grouped the studies into "low risk," "high risk," or "unclear risk." Studies were considered low risk of bias if they sufficiently addressed all the elements of selection, performance, attrition, and reporting bias. Studies with insufficiently addressed outcomes were considered to bear a high risk of bias, while studies where a clear judgment could be made, were considered to have an unclear risk of bias. The risk of bias graph is presented in Figure 1.

**Figure 2:**
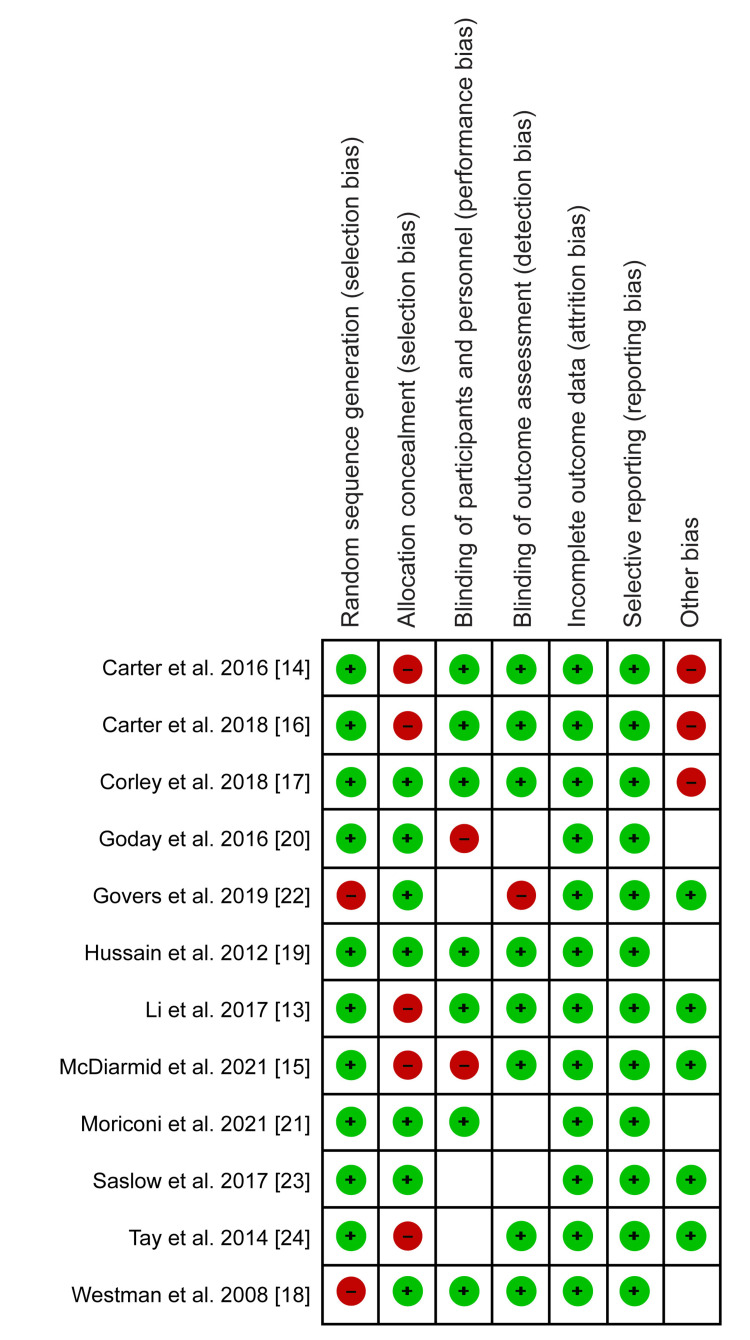
Risk of bias summary. Source: Li C et al. (2017) [13], Carter S et al. (2016) [14], McDiarmid S et al. (2021) [15], Carter S et al. (2018) [16], Corley BT et al. (2018) [17], Westman EC et al. (2008) [18], Hussain TA et al. (2012) [19], Goday A et al. (2016) [20], Moriconi E et al. (2021) [21], Govers E et al. (2019) [22], Saslow LR et al. (2017) [23], Tay J et al. (2014) [24]

The risk of bias summary is presented in Figure 2.

Synthesis of Results

Meta-analyses showing the pooled effect of outcomes were carried out using RevMan software. A random effect was preferred in the analysis since it sufficiently addressed the expected heterogeneity. The heterogeneity was measured using the I2 statistics, where heterogeneity scores below 50%, 51-70%, and above 70% were deemed low, moderate, and high, respectively. The statistical power of the present meta-analysis was also improved by choosing a CI of 95%. Additionally, statistical significance was assessed as p<0.005. All the meta-analysis results were presented in forest plots.

Results

Study Selection

**Figure 3:**
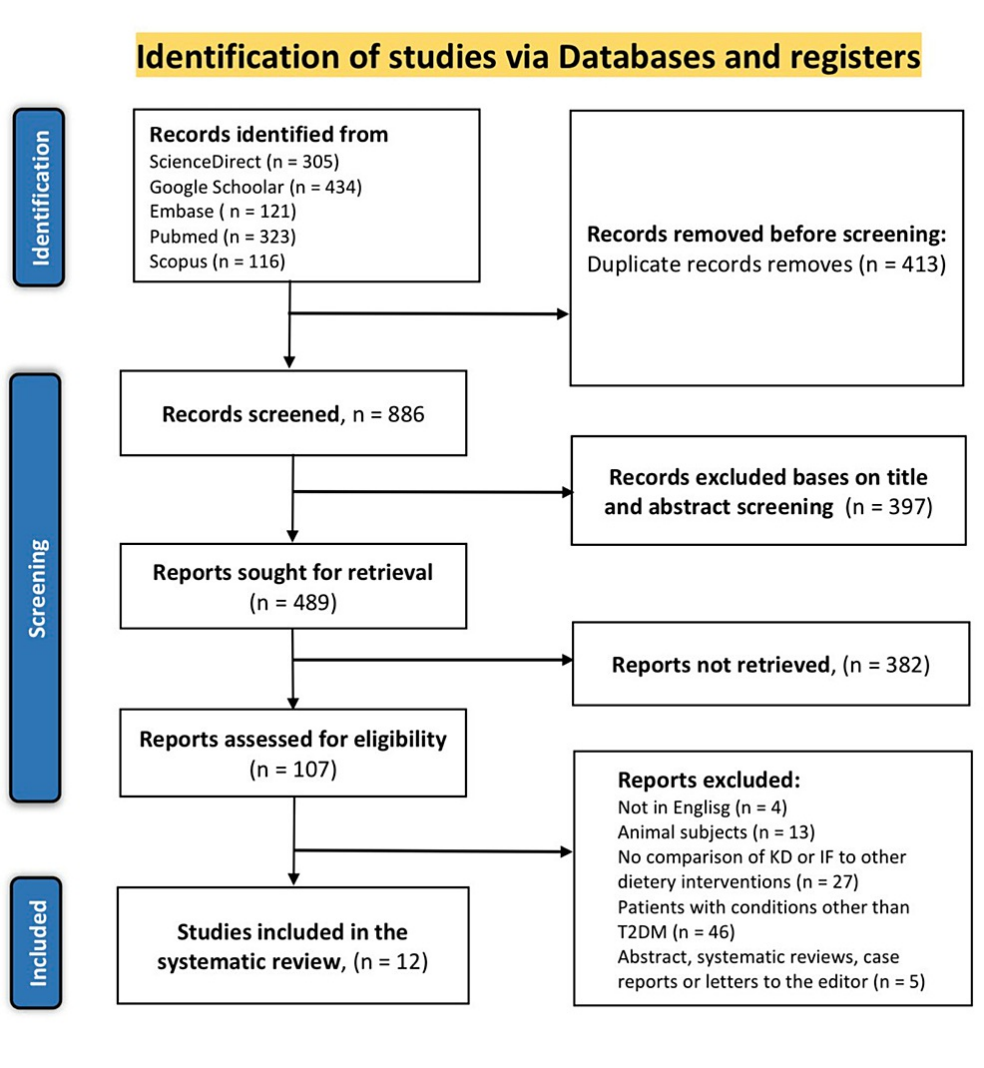
PRISMA flow diagram of the literature search results. PRISMA: Preferred Reporting Items for Systematic Reviews and Meta-Analyses.

The detailed literature search through the mentioned electronic databases resulted in 1299 articles. The 1299 records were then screened for duplicates, of which 413 were excluded. The remaining 886 records then had their titles and abstracts filtered, of which 397 did not meet the screening criteria, and 382 were not retrieved. The 107 retrieved articles were assessed using the eligibility criteria outlined for the present study, and only 12 articles met the inclusion criteria. The other 95 articles were excluded as follows: 13 included animal subjects, 27 did not compare either KD or IF with other dietary interventions, 43 compared either KD or IF in patients with diseases or conditions other than T2DM, four were published in languages other than English, and five were either abstracts without full articles, systematic reviews and meta-analyses, letters to the editor or case reports. The study selection results are presented in the PRISMA flow diagram shown in Figure 3.

Study Characteristics

Study characteristics are described in Table 1.

**Table 1:** Study characteristics. Source: Li C et al. (2017) [13], Carter S et al. (2016) [14], McDiarmid S et al. (2021) [15], Carter S et al. (2018) [16], Corley BT et al. (2018) [17], Westman EC et al. (2008) [18], Hussain TA et al. (2012) [19], Goday A et al. (2016) [20], Moriconi E et al. (2021) [21], Govers E et al. (2019) [22], Saslow LR et al. (2017) [23], Tay J et al. (2014) [24] RCT: Randomized controlled trial: CER: Continuous energy restriction; IER: Intermittent energy restriction; HBA1c: Glycated hemoglobin; ILED: Intermittent low-energy diet; CLED: Continuous low-energy diet; LCKD: Low-carbohydrate, ketogenic diet; LGID: Low-glycemic, reduced-calorie diet; LCD: Low-calorie diet; VLCKD: Very low-carbohydrate ketogenic diet/Very low-calorie ketogenic diet; ERD: Energy restricted diet; ADA: American Diabetes Association; HC: High carbohydrate.

Author ID	Study Design	Country	Participants	Intervention diet	Control diet	Main Outcomes
Li C et al. (2017) [13]	RCT	Germany	46 (25-75 years)	An initial 2-day pre-fasting while receiving a low-calorie diet (1200kcal) followed by a 7-day fasting period while receiving a daily energy intake of 300kcal	Mediterranean diet	A 3.5 ± 4.5 kg mean body weight was recorded in the fasting group, while a mean decrease of 2.0±4.8kg was recorded in the control group. An insignificant difference in HBA1c was recorded in the fasting group compared with the control group (-1.2±1.1 vs.-0.2±0.8, p=0.70).
Carter S et al. (2016) [14]	RCT	Australia	63 (30 male and 33 female)	2-day per week energy restriction (1670-2500kj/day) followed by 5-day habitual eating.	Continuous energy restriction for 7 days (5000-6500kJ/day).	A significant weight change was observed over time (99 ± 14kg to 93 ± 13kg; P<0.001); however, no significant change was recorded in the CER and IER group (102 ± 17kg to 94 ± 13kg vs. 100 ± 20kg to 92 ± 14kg, respectively; P=0.7). A significant HBA1c decrease with time was recorded (-0.7 ± 0.9% P<0.001); however, the difference was insignificant with dietary interventions (-0.8% ± 1 vs. -0.6% ± 0.8% for CER and IER, respectively; P=0.3)
McDiarmid S et al. (2021) [15]	RCT	United Kingdom	79 (37 female and 42 males; mean age 55.5 ± 11.3 years	39 Patients subjected to ILED followed a 2-day Optifast and 5-day controlled Mediterranean diet for 28 weeks.	40 patients subjected to the CLED were recommended to follow an optifast low-energy diet for 8 weeks (3430 kJ)/day) followed by the reintroduction of 1000 kcal (4184 kJ) to 1500 kcal (6276 kJ)/day energy foods over 4 weeks	Over a 28-week follow-up, the mean weight loss observed in the ILED and CLED groups were -5.1 (-7.1, -3.2) and -6.2(-8.1, -4.3), respectively. The HBA1c mean changes observed were -7.9 (-11.5, -4.2) and -8.5 (-12.0, -4.9) for ILED and CLED groups, respectively.
Carter S et al. (2018) [16]	RCT	Australia	137 (77 males and 60 females; mean age 61.0 (9.1) years)	70 patients were subjected to an IER diet (500-600kcal/day for 2 days a week and 5 days of usual eating)	67 patients followed the CER diet (1200-1500kcal/day)	The body weight reduction was insignificant between the two groups (-5.0 vs. -6.8 kg for CER and IER, respectively). No significant difference was observed in HBA1c (-0.5% vs. -0.3% for CER and IER groups, respectively).
Corley BT et al. (2018) [17]	RCT	New Zealand	41 (15 female and 26 males)	19 patients followed the IF diet with non-consecutive fasting days	18 patients followed the diet with consecutive fasting days	After 12 weeks, the non-consecutive and consecutive showed an insignificant difference in body weight (109.8 (20.3) to 106.2 (20.1) vs. 108.7 (20.4) to 105.6 (19.9) kg). An insignificant change in HBA1c was recorded (8.2 (1.3) to 7.5 (1.5) vs. 8.4 (1.8) to 7.8 (1.8) for non-consecutive and consecutive groups, respectively).
Westman EC et al. (2008) [18]	RCT	United States of America	50 (37 female and 13 males)	29 patients subjected to the LCKD group received a daily dietary intake of <20g carbohydrates daily.	21 patients in the LGID group received approximately 55% energy from carbohydrates daily.	A larger HBA1c decrease from the baseline was recorded in the LCKD group than LGID group (8.8 ± 1.8% to 7.3 ± 1.5%) vs. (8.3 ± 1.9% to 7.8 ± 2.1%), respectively. Patients in the LCKD group recorded a higher weight loss than LGID (108.4 ± 20.5 kg to 97.3 ± 17.6 kg vs. 105.2 ± 19.8 to 98.3 ± 20.3 kg, respectively).
Hussain TA et al. (2012) [19]	RCT	Kuwait	102 T2DM patients	78 patients in the LCKD group were restricted to a carbohydrate intake of approximately 20g daily.	24 patients were subjected to LCD	Patients in the LCKD group recorded a significant change in weight than patients in the LCD group (104.01 ± 18.89 to 91.56 ± 17.45 kg vs. 95.71 ± 9.56 to 89.02 ± 5.97 kg, respectively). The LCKD group showed a significant change in HBA1c than the LCD group. An insignificant difference in BMI values was observed (36.31 ± 2.63 to 33.87 ± 2.75 kg/m^2^ vs. 39.84 ± 6.40 to 35.05 ± 5.90 kg/m^2^ for LCKD and LCD groups, respectively.
Goday A et al. (2016) [20],	RCT	Spain	89 (31 men and 58 women; mean age 54.53 (8.37) years.	45 patients were subjected to a VLCKD (600-800kcal low-calorie diet, low in carbohydrates (<50g from vegetables every day) and lipids (10 g olive oil per day)	44 patients followed LCD (500-1000kcal/day calorie restriction)	A larger weight loss was recorded among patients in the VLCKD group than in the LCD group (91.5 (11.4) to 76.8 (9.1) kg vs. 90.0 (11.3) to 84.95 (13.6) kg, respectively). A significant decrease in HBA1c was observed in the VLCKD group (6.9 (1.1) to 6.0 (0.7) %, p <0.00001) while the change was insignificant in LCD group (6.8 (1.0) to 6.4 (0.8) %, p = 0.1453).
Moriconi E et al. (2021) [21]	Retrospective observational study	Italy	30 (14 males and 16 females; aged 35-75 years).	15 patients were subjected to VLCKD (required <800kcal total energy and 1.2-1.5kg protein intake in the first phase while in the second phase, the patients were introduced to conventional food)	15 patients were subjected to LCD (daily energy intake of (500-1000 kcal. 30% calories from fat, 20-25% from protein, and 40-50% from carbohydrates.	A 3kg weight loss was observed among patients in the VLCKD group at 3 and 12 months, while an insignificant change was recorded in the LCD group. The VLCKD and LCD groups showed a decrease of 0.69 ± 0.65% and 0.42 ± 0.01% in HbA1c.
Govers E et al. (2019) [22]	Retrospective study	Netherlands	344 (151 male and 193 females; mean age 62.1(10.8) years)	110 patients were subjected to a 6*6 VLCKD	123 patients were subjected to LCD, and 111 were subjected to ERD	The 6*6 and LCD groups showed similar reductions in HBA1c at 3,6, and 12 months. At 3, 6, and 12 months the weight loss observed in the HBA1c was 2.3, 3.0, and 2.7 kg more than the LCD group and 3.9,3.7, and 3.5 kg more than the ERD group.
Saslow LR et al. (2017) [23]	RCT	United States	25 (10 male and 15 females)	12 patients followed the ad libitum VLCKD (20-50 g reduction in carbohydrate intake)	13 patients received the ADA “create your plate” diet	At 16 and 32 weeks, the VLCKD group showed significant reductions in HBA1c than the control group (-0.9% vs. -0.5% and -0.8% vs. -0.3%, respectively). A significantly larger weight loss was recorded in participants randomized to the VLCKD group than in the control group at 16 and 32 weeks (-7.8 vs. -4.2 and -12.0 vs. -2.5kg, respectively).
Tay J et al. (2014) [24]	RCT	Australia	115 (66 males and 49 females)	46 patients that completed the trial received a VLCKD (The energy intake was 14% carbohydrates, 28% protein, and 58% total fats	47 patients that completed the trial received an HC diet (The energy intake was 53% carbohydrates, 17% protein, and <30% total fat.	A greater HBA1c reduction was observed in the VLCKD group for patients with baseline HBA1c > 7.8%. The VLCKD group recorded a higher BMI decrease than the HC group (4.0 (2.0) vs. -4.0 (1.8), respectively).

Weight Control

**Figure 4:**
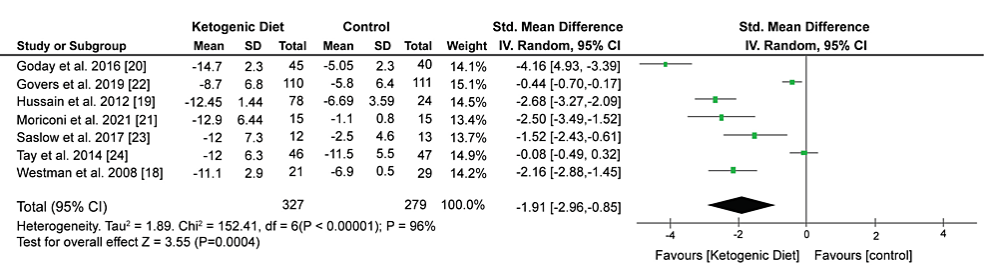
Forest plot showing the effect of KD on body weight. KD: Ketogenic diet. Source: Westman EC et al. (2008) [18], Hussain TA et al. (2012) [19], Goday A et al. (2016) [20], Moriconi E et al. (2021) [21], Govers E et al. (2019) [22], Saslow LR et al. (2017) [23], Tay J et al. (2014) [24]

Pooled data from five studies have shown that the KD significantly reduced body weight compared with other control diets (SMD: -1.91 kg; 95% CI; -2.96 kg, -0.85 kg; P = 0.0004, I2 = 96%). Figure 4 shows the Forest plot of the effect of KD on body weight.

**Figure 5:**
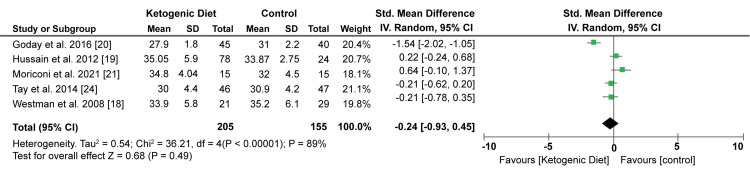
Forest plot showing the effect of KD on BMI. KD: Ketogenic diet. Source: Westman EC et al. (2008) [18], Hussain TA et al. (2012) [19], Goday A et al. (2016) [20], Moriconi E et al. (2021) [21], Tay J et al. (2014) [24]

However, the KD showed an insignificant reduction in BMI compared with control diets (SMD: -0.24; 95% CI; -0.93, 0.45; P = 0.49, I2 = 89%). Figure 5) shows the Forest plot of the effect of KD on BMI.

**Figure 6:**
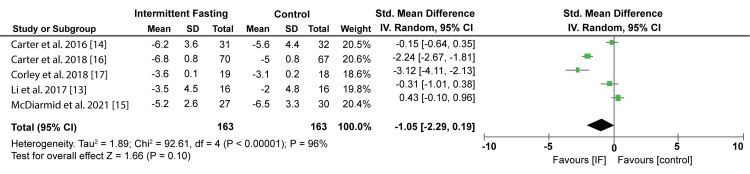
Forest plot showing the effect of IF on body weight. IF: Intermittent fasting. Source: Li C et al. (2017) [13], Carter S et al. (2016) [14], McDiarmid S et al. (2021) [15], Carter S et al. (2018) [16], Corley BT et al. (2018) [17]

The pooled effect of IF also showed that body weight decreased by 1.05 kg. However, a comparison with other control interventions has shown that the difference was insignificant (p = 0.10). Figure 6 shows the Forest plot of the effect of IF on body weight.

**Figure 7:**
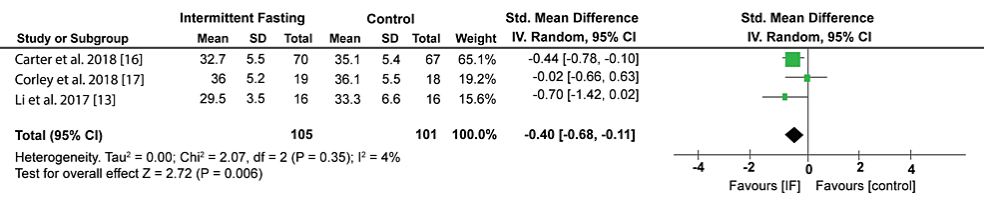
Forest plot showing the effect of IF on BMI. IF: Intermittent fasting. Source: Li C et al. (2017) [13], Carter S et al. (2018) [16], Corley BT et al. (2018) [17]

A meta-analysis of data from three studies has also shown that IF has a significant reduction in BMI than control diets (SMD: -0.40; 95% CI; -0.68, -0.11; P = 0.006, I2 = 4%). Figure 7 shows the Forest plot of the effect of IF on BMI.

Glycemic Control

**Figure 8:**
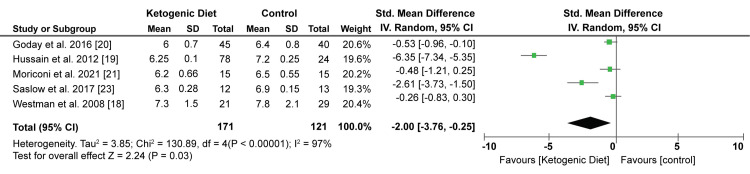
Forest plot showing the effect of KD on HBA1c. KD: Ketogenic diet. Source: Westman EC et al. (2008) [18], Hussain TA et al. (2012) [19], Goday A et al. (2016) [20], Moriconi E et al. (2021) [21], Saslow LR et al. (2017) [23]

Five studies evaluated the impact of KD on HBA1c percentage. A meta-analysis of data from these studies has shown that KD significantly decreases the HBA1c compared with control intervention diets (SMD: -2.00%; 95% CI; -3.76, -0.25; P = 0.03, I2 = 97%). Figure 8 shows the Forest plot of the effect of KD on HBA1c.

**Figure 9:**
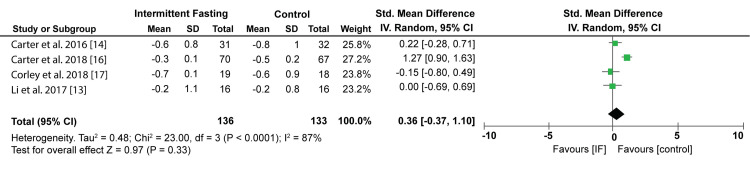
Forest plot showing the effect of IF on HBA1c IF: Intermittent fasting. Source: Li C et al. (2017) [13], Carter S et al. (2016) [14], Carter S et al. (2018) [16], Corley BT et al. (2018) [17].

On the other hand, the pooled results from four studies show that IF had an insignificant impact on HBA1c compared to control intervention diets (SMD: 0.36%; 95% CI; -0.37, 1.10; P = 0.33, I2 = 87%). Figure 9 shows the Forest plot of the effect of IF on HBA1c.

Discussion

The present study evaluated the role of IF and KD in glycemic and weight control among patients with T2DM. The analysis carried out in the current research has shown that KD significantly reduced HBA1c and body weight compared with control interventions. However, the meta-analysis reported an insignificant impact on BMI values. On the other hand, IF showed a decrease in HBA1c and body weight. However, when compared to control interventions, the difference was statistically insignificant. The analysis of the three included studies also showed that IF significantly reduces the BMI values compared to the control interventions.

We initially hypothesized that IF would significantly decrease the body weight, BMI, and HBA1c percentage in patients with T2DM. However, our results have shown that IF has a similar effect on HBA1c as the control interventions, which constitute mainly continuous energy restriction (CER). These results are reinforced by a previous meta-analysis of six randomized studies, which reported that IF showed an insignificant decrease in HBA1c compared with the control arm (SMD = -0.11%; 95% CI: -0.38%, 0.17%) [25]. Similarly, a previous randomized trial reported that after 20 weeks of dietary interventions, no difference in HBA1c values was observed between the IF and standard diet groups [26]. However, the study reported that the target HBA1c was achieved mostly by patients in the IF group. Wing RR et al. also claimed that IF had a similar effect on HBA1c as the low-calorie diet [27]. Even though our results show an insignificant impact on HBA1c, evidence in some of the included studies has shown that HBA1c levels are affected by the duration of intervention. For example, Carter S et al. [14] reported that the HBA1c was comparable with the interventions, but a significant decrease in HBA1c with time was recorded (-0.7 ± 0.9% P<0.001). Other measures, such as fasting glucose, are also vital in understanding the impact of IF on glycemic control. Corley BT et al. reported that non-consecutive and consecutive fasting has no significant impact on fasting glucose (7.9 (1.7) vs. 6.9 (2.1) mmol/l, P = 0.21) [17]. Similarly, Wing RR et al. [27] showed that patients following the intermittent, very-low-calorie, and low-calorie diets had comparable fasting glucose outcomes (9.78 + 3.28 vs. 9.28 + 3.67, respectively).

Additionally, our meta-analysis shows that IF reduced the body weight of patients with T2DM by 1.05 kg. These results are supported by a recent meta-analysis which recorded a significant weight loss of 1.89 kg [25]. Similarly, a three-phase observational study evaluating the impact of IF in adult patients with T2DM also recorded a significant weight loss of 1.395 kg during the second phase (two-week intervention) [28]. The study also shows that during the third phase (two-week follow-up), a 1.12 kg weight loss was recorded. However, the mean difference between the first and third phases showed that the weight loss was insignificant (p = 0.08). This insignificant change can be attributed to the patients returning to a normal diet in the third phase. A much greater weight loss in T2DM patients has been reported in a study that applied three different isocaloric diet regimens [29]. Results of that study showed that the patients had lost 6% of their initial body weight at the end of 12 weeks (6.4 + 4.6 kg; P < 0.001). However, after 18 months, 85.2% of the patients regained >1kg, and no weight loss was recorded thereafter. The great weight loss recorded in that study can be explained by the fact that it included only male patients. According to a more recent systematic review and meta-analysis, men were found to lose more weight than women [30]. This substantial decrease in weight can be related to the fact that men contain more metabolically active tissues than women, thus contributing to a higher resting energy expenditure and greater weight loss [31,32].

Our results have also shown that IF significantly decreases the BMI among T2DM patients. These results are supported by a previous randomized crossover study which reported that IF significantly reduced BMI. The study showed that patients who took two meals per day had a significantly higher reduction in BMI than patients that took six meals every day (-1.23 vs. -0.82kg/m2, p<0.001) [33]. Evidence also shows that IF has an effect on the medication effect score (MES). A decrease in MES corresponds to a reduction in medication dosages, while an increase in MES corresponds to an increase in diabetes medications. According to Carter S et al. [16], the total mean MES decreased with time. However, no significant difference was observed between the intermittent energy restriction (IER) and CER groups (-0.6 (0.1) vs. -0.3 (0.1), respectively; p=0.1). Similarly, a 2016 study showed that total mean MES decreased with time, but there was no significant difference between the treatment groups (-0.4 ± 0.6 vs. -0.4 ± 0.5 for CER and IER, respectively) [14]. A randomized trial also reported that after 52 weeks, 15% of patients in the intermittent low-energy diet group (ILED) and 43% of patients in the continuous low-energy diet group (CLED) had a reduction in diabetes medication. However, the total mean MES was not significantly different between the treatment group (0.1 (-0.1, 0.4) vs. -0.5 (-0.8, -0.2) for ILED and CLED, respectively) [15].

Even though we have shown that IF has promising glycemic and weight control outcomes, it is subject to side effects. Evidence shows that the most occurring side effect of intermittent fasting is hypoglycemia. Corley BT et al. evaluated the risk of developing hypoglycemia among T2DM patients following the 5:2 IF protocol and found that over a 12-week observation period, 53 hypoglycemic events were recorded in 15 participants [17]. The study's results also showed that the risk of developing hypoglycemic events was twice greater during the fasting period (RR 2.05 (95% CI 1.17-3.52); P=0.013). However, no significant difference was observed in hypoglycemic events between the consecutive and non-consecutive fasting groups (relative risk (RR) 1.54 (95% CI: 0.35-6.11); P = 0.51). Similarly, a more recent study evaluating the risk of hypoglycemia during Ramadhan fasting in diabetic patients reported that 13.3% with T2DM had hypoglycemic events [34]. However, the analysis in this study showed that patients with T1DM were significantly at a higher risk of developing hypoglycemia than patients with T2DM (p<0.0001). This study also reported that substantially higher age was recorded for patients with hypoglycemia and T2DM. Apart from hypoglycemia, other adverse events, including diarrhea, fatigue, headaches, constipation, and dizziness, have also been associated with IF. McDiarmid S et al. [15] reported six diarrhea cases, three fatigue cases, one headache case, two constipation cases, and one nausea/vomiting case for patients in the ILED group. Li C et al. [13] also reported that three participants reported headaches during fasting, while one patient reported slight dizziness during the later fasting days.

The current study also hypothesized that KD would significantly reduce HBA1c, body weight, and BMI. This hypothesis was supported since the pooled effect showed that KD significantly reduced the body weight of patients with T2DM by 1.91 kg. These results are consistent with our previous meta-analysis, which reported a 2.67 kg weight reduction for patients following the KD protocols [35]. Similarly, a previous meta-analysis of four randomized trials reported a significant weight loss among T2DM patients following the KD protocol than patients subjected to other diets (SMD: -4.26kg; 95% CI; -6.88 kg, -1.63 kg; p = 0.001; I2 = 81%) [36]. Other consistent results have been reported in a meta-analysis of seven randomized trials, which recorded a significant weight loss of 7.78 kg for patients in the KD group [37]. The significant weight loss recorded by patients following the KD can be attributed to various reasons. First, KD involves eating foods with more fat and protein, which have greater satiety than carbohydrates, partly because they have longer digestion time, thus slowing down the stomach release, and partly because they induce more ketone, which lowers the patients’ appetite [7,38]. These effects have been described as pleasant since the patients feel they can control their appetite, which is new to them. Secondly, the high protein intake attained during the KD is associated with better muscle mass preservation, stimulating energy expenditure and causing more weight loss [22]. Some contradictory results have been presented in some of the included studies. Tay J et al. [24] reported an insignificant difference in weight loss after a 24-week follow-up between the very low-carbohydrate and high-carbohydrate groups (-12.0 (6.3) kg vs. -11.5 (5.5) kg, respectively; p=0.57) [24]. The results of the current meta-analysis have also shown that KD has an insignificant difference compared with control diets in BMI reduction (p=0.49). These results are reinforced by our previous meta-analysis, which reported an insignificant difference in BMI between patients following the KD and other dietary interventions (SMD: -0.31 kg/m2; 95% CI; -0.81 kg/m2, 0.20 kg/m2; P=0.23, I2 = 84%) [35]. Choi YJ et al. also reported that KD had an insignificant reduction in BMI compared with control interventions (SMD: -0.63 kg/m2; 95% CI; -1.31 kg/m2, 0.08 kg/m2; P = 0.08, I2 = 87%) [37]. However, a previous study by Li S et al. [39] reported that after a 12-week follow-up, patients in the KD group recorded a significantly lower BMI than patients subjected to a diabetic diet (26.21 ± 5.74 vs. 29.42 ± 5.97, respectively). The significant change recorded in this study can be explained by the fact that KD stimulated different starvation degrees. Thus, the body uses the energy supplied by the ketone body.

Additionally, the results of the current study have supported the initial hypothesis by showing that KD significantly reduced the HBA1c. The reduction of 2% in HBA1c reported in the present study is consistent with the results of previous meta-analyses, which showed a significant decrease of 1.02% in HBA1c [36]. The reduction in HBA1c can be explained by the fact that KD also reduced body weight among patients with T2DM. Another important measure for glycemic control discussed in previous studies is fasting glucose. Goday A et al. [20] reported that patients that followed the KD protocol achieved a significant reduction in fasting glucose than patients that followed the low-calorie diet protocol (108.9 (20.4) mg/dL vs. 123.3 (24.3) mg/dL). These results are reinforced by a previous study that reported that KD lowered the fasting insulin, thus stabilizing blood glucose and alleviating any blood glucose fluctuations in T2DM patients [40]. Despite the current meta-analysis showing that KD offers better weight and glycemic control outcomes, evidence has shown that it has some side effects. Westman EC et al. reported that the most common adverse effects recorded in their study were headache and constipation, with an occurrence rate of 53.1% among patients in the KD group [18]. Other side effects, such as diarrhea, insomnia, and back pain, accounted for 40.6%, 31.2%, and 34.4% of patients in the KD groups. Tay J et al. [24] also reported that five patients in the KD group experienced musculoskeletal ailments, two reported cases of GI disorders, including constipation and diverticulitis, and one patient was diagnosed with prostate cancer. Due to these side effects, dietitians are required to provide appropriate guidance to the patients.

Limitations

The primary limitation of the current study is the high heterogeneity recorded during meta-analysis. However, high heterogeneity is expected since the dietary interventions used in each study were not standardized. Additionally, the varying sample sizes recorded in each study could have contributed to the high heterogeneity. Due to this high heterogeneity, the results of our meta-analyses should be interpreted with caution; however, most of the studies used in the review were of good methodological quality, thus minimizing the publication bias. The other limitation of the current study is based on the eligibility criteria, which allowed the inclusion of studies published in English only. This specification may have led to omitting some relevant information that would have otherwise been used to improve the statistical power of our meta-analysis. The current study also did not categorize the IF protocols used in each study, therefore; making it difficult to understand the effectiveness of different IF protocols on weight and glycemic control.

## Conclusions

The increasing prevalence of T2DM across the world requires effective primary-care treatments. Some potential methods to reduce the incidence and severity of T2DM include IF and KD. These methods can manage T2DM through weight loss and glycemic control. The current study has shown that IF reduces both body weight and HBA1c; however, compared with control diets consisting mainly of CER diets shows an insignificant difference. The pooled results have also suggested that IF significantly reduced BMI compared to control interventions. On the other hand, the KD diet has proved to be an effective strategy in reducing body weight and HBA1c. Despite the benefits of IF and KD on weight and glycemic control, it is essential to note that the regimens have potential side effects. Therefore, the interventions should be used cautiously and under the health professional’s supervision. Evidence has also shown that IF studies, including male patients, show higher weight loss; however, more research is required to compare the effect of gender on weight loss. Based on our result, we can suggest that IF and KD are reliable methods for weight loss; however, the high heterogeneity recorded makes it challenging to compare the effect of the two diets on weight loss and glycemic control. Future research should be carried out to directly compare the effectiveness of KD and IF on weight and glycemic control.
